# Retrospective Study on Risk Factors and Short-Term Outcome of Horses Referred for Colic from 2016 to 2022

**DOI:** 10.3390/vetsci9100545

**Published:** 2022-10-03

**Authors:** Paola Straticò, Vincenzo Varasano, Adriana Palozzo, Giulia Guerri, Gianluca Celani, Ortensia Revelant, Lucio Petrizzi

**Affiliations:** Faculty of Veterinary Medicine, University of Teramo, Località Piano d’Accio, 64100 Teramo, Italy

**Keywords:** colic, equine, laparotomy, surgery, survival, predisposing factors

## Abstract

**Simple Summary:**

Colic syndrome is the most common out-of-hours call for equine practitioners. In most cases, medical treatment at home is effective for the resolution of the disorders. In 20% of cases, hospital intensive care is required. Several factors have been addressed as predisposing to the disorder. Cardiovascular and hemodynamic variables are the most reliable indicators of the severity of the condition and, thus, are important prognostic factors. The aim of the study was to highlight the predisposing factors and best prognostic clinical signs for discharge in a cohort of horses referred for colic syndrome. Medical records from 2016 to 2022 were examined. One hundred and fifty-seven horses were included in the study. Month and time of admission were the main predisposing factors. Cardiovascular variables (heart rate on admission and after 3–6 h, packed cell volume, capillary refill time, and mucosal membranes) and the presence of gastric reflux were negatively associated with discharge as well as time to standing after surgery. The results from this retrospective study may help the clinician in evaluating prognosis, deciding on the best treatment, and adequately informing the owner of the rates of success.

**Abstract:**

(1) Background: colic syndrome is a multifactorial life-threatening condition in equids. Awareness of predisposing and prognostic indicators is useful to adequately inform the owner of the outcome and plan the best treatment. The aim of the study was to identify the variables associated with short-term survival in a cohort of horses referred for colic to a veterinary teaching hospital; (2) Methods: medical records of horses referred for colic from 2016 to 2022 were retrospectively reviewed. Univariate and multivariate regression models were built to assess the odds ratios of discharge from the hospital, both on the whole sample and in those patients undergoing surgery; (3) Conclusions: Month, time of admission, cardiovascular variables (heart rate on admission and after 3–6 h, packed cell volume, capillary refill time, and mucosal membranes), and presence of gastric reflux were significantly associated with discharge in the univariate regression in all horses and in those surgically treated. In the multivariate regression model, heart rate, packed cell volume, and capillary refill time showed significant association with the outcome in the whole sample. Although comparison between this study and previous studies is difficult due to the number and type of variables included, cardiovascular variables and markers of hypovolemia were also shown to be associated with outcome.

## 1. Introduction

Abdominal pain in horses is one of the most common causes of emergency presentation to equine veterinary practice, accounting for approximately 30% of all emergency calls for equine practitioners/surgeons [1,2]. It may be caused by a variety of conditions, ranging from simple spasmodic colic to strangulating disorders [3], as well as deep ulcers or peritonitis [4]. Even though in most cases simple medical management is efficient for the resolution of clinical signs, 8–20% of cases are critical and require hospitalization for intensive care, either medical or surgical treatment, or euthanasia [5,6].

Several predisposing factors have been identified with the onset of colic syndrome, such as patient-related (age, breed, and gender) and management-related factors (changes in diet, exercise, housing, and intestinal parasites) [6,7,8,9,10,11,12].

Association of seasonality, but not barometric pressure [13], with colic incidence is recognized [9,14,15,16], but the results are different according to the geographical location, with some studies addressing hot months as more predisposing to the occurrence of symptoms [8] while some others showing cold weather as more related to them [17].

Hospitalization of horses with colic signs requires emergency transportation to the referral practice. During transportation, dehydration may worsen due to stress, sweating, and absence of adequate fluid therapy. Transportation is also one of the main causes of stress for horses, as it is responsible of disruption of homeostasis [18,19,20,21]. Therefore, distance from and time required to reach the referral practice may worsen the clinical conditions of the horse and act as a predisposing factor.

Although definitive diagnosis may be difficult to achieve without an exploratory laparotomy, the equine clinician is limited in the amount of information they can provide to a horse owner regarding prognosis and outcome without having a definitive diagnosis. Therefore, having physiologic variables—such as cardiovascular parameters, hypovolemic markers, and abdominal borborygmic which are measures that provide the clinician with information regarding patient status—may impact prognosis and outcome [3]. Although outcome is related to a variety of physiologic variables, cardiovascular parameters, hypovolemic markers, and abdominal borborygmi are identified as strictly correlated to it [5,22].

The clinical conditions of the horse on admission and its insurance status, owner’s compliance and finances, and the availability of medical facilities are important aspects that may influence the decision-making process for the treatment [23,24,25]. Five general criteria have been adopted to help the surgeon in deciding whether a medical or surgical treatment must be pursued: pain response to analgesia, presence of gastric reflux, cardiovascular conditions, results of peritoneal tap, and findings at rectal palpation [14].

A variety of studies investigated risk and prognostic factors, sometimes with conflicting results, likely related to differences in horse population and geographical influences [26,27]. The aim of this study was to analyze the population of horses referred for colic syndrome in one referral practice in the center of Italy and to identify risk factors related to the survival and discharge from the hospital. Clinical parameters that are potentially associated to non-survival were also investigated. We hypothesized that variables associated with the outcome could be identified which could help the surgeon in the decision-making process when deciding the best approach for the clinical case.

## 2. Materials and Methods

Clinical records of horses referred for colic syndrome to the University of Teramo between January 2016 and May 2022 were reviewed. Cases with abdominal pain for reasons unrelated to the gastrointestinal tract were excluded. For horses that were admitted more than once within twelve months, only the first admission was considered. For horses that underwent more than one laparotomy during the same hospitalization, only the first was considered.

Patient data that were retrieved from medical records were: age, gender (mare/stallion/gelding), breed, progression of clinical signs before referral (improved/deteriorating/no progression), medication received before referral (none/NSAIDs/alpha-2-agonists/spasmolytics/crystalloids), distance to be covered to reach the referral practice (distance from the surgical facility-km: close/medium/far, according to the distribution of data), time of the day and season of admission (morning/afternoon/night; January-March/April-June/July-September/October-December), and type of treatment attempted (medical/surgical). On admission the following data were recorded: heart rate (HR, beats/min), rectal temperature (°C), mucous membrane color and capillary refill time (normal/pale/hyperemic without toxic line/hyperemic with toxic line/cyanotic/brick red; CRT, seconds), mentation (depressed/stuporous/agitated/bright), severity of pain (none/mild/moderate/severe) [28], presence of gastric reflux, abdominal distension (none/bilateral/right flank/left flank), gut sounds on auscultation (normal/hyperkinesia/hypokinesia/akinesia), packed cell volume (PCV, %), total protein (TP, gr/dL), HR 3 h after admission or after reaching the standing position after surgery (beats/min), and duration of hospitalization (days). The outcome was dichotomized as discharged/non-discharged. For those horses that underwent laparotomy, the following data were retrieved from surgery and the anesthetic records: anesthetic protocol, duration of anesthesia and surgery (minutes), time to standing (minutes) and recovery score [29], type of lesion (ischemic/non-ischemic), and occurrence of complications (if any/the type of complication).

All data were collected and stored in an Excel worksheet and analyzed with free statistical software [30]. Categorical variables were reported as frequency counts (percentage of total), whereas continuous variables were assessed for normality with the Shapiro–Wilk test and visual evaluation of the histograms and expressed either as mean ± standard deviation (if normally distributed), or median and range (if not normally distributed).

A univariate logistic regression was applied to highlight potential association between the independent variables and the outcome, first within all the horses included in the study and later only in those undergoing surgery. Variables that showed evidence of association (*p* < 0.2) in the univariate logistic regression model were selected for a multivariable logistic regression analysis. The goodness-of-fit of the model was assessed with the Akaike information criterion (AIC). The variables were considered significantly associated with the outcome for *p* < 0.05. The results of both the uni- and multivariate regression models are shown as odds ratio (OR) and 95% confidence interval (CI).

## 3. Results

One-hundred and fifty-seven clinical records were selected for the research: 82/157 (52%) were treated surgically and 75/157 (48%) received medical treatment.

Gender, age, and body weight for horses that received either a surgical or medical treatment are shown in Table 1.

Patients selected were represented by Warmbloods (54/157, 34.5%), Western riding horses (38/157, 24%), Thoroughbreds (31/157, 20%), Friesians (7/157, 4.5%), Spanish horses (3/157, 2%), and Others (24/157,15%). In the category “Others”, trotters, ponies, donkeys, and mules are included.

When the whole sample of horses was examined, the signalment findings shown in Table 2 were observed. No statistical difference for the signalment variables was found between horses discharged and non-discharged (body weight, age, gender, and breed; *p* > 0.05).

On admission progression of clinical signs as described from the owner/trainer, mentation, CRT, PCV, gastric reflux, and HR were statistically different between discharged and non-discharged horses (Table 3) (*p* < 0.05). HR 3–6 h after admission or surgical treatment (if this was attempted straight after admission) was also different among the two groups (*p* < 0.05).

The variable “Distance from the surgical facility (km)” was categorized according to the distribution of data as follows: “Close”, if the distance was less than 19 km, “Medium”, if the distance ranged between 19 and 179 km, and “Far” if the distance was more than 179 km.

In the variable “Medication received before referral”, the category “Mixed” was represented by the following treatments/associations:

- spasmolytics, NSAIDs, crystalloids, and enteral fluid therapy
- spasmolytics, NSAIDs, gastroprotectants, opioids, and metoclopramide
- spasmolytics and enteral fluid therapy
- NSAIDs and enteral fluid therapy
- NSAIDs and alpha-2-agonists
- spasmolytics, NSAIDs, and alpha-2 agonists
- spasmolytics and alpha-2-agonists
- spasmolytics and NSAIDs
- antibiotics and corticosteroids
- NSAIDs and crystalloids
- NSAIDs, alpha-2-agonists, and metoclopramide
- NSAIDs, alpha-2-agonists, and crystalloids
- alpha-2-agonists and crystalloids
- NSAIDs, crystalloids, and enteral fluid therapy
- corticosteroids, NSAIDs, and alpha-2-agonists

When non-discharged, the horses were euthanized in 52/55 cases (96%); six of these received intraoperative euthanasia. In 3/55 cases spontaneous death was recorded.

The univariate logistic regression between the independent variables recorded on admission and the outcome is shown in Table 4. HR on admission, PCV, and CRT were positively associated with the odds ratio of non-discharge; cyanotic and brick red mucosal membrane color had increased odds ratio of non-discharge compared with the reference category (normal mucosal membrane color). Admission between October and December was negatively associated with the outcome.

Those variables that showed a *p* ≤ 0.2 in the univariate model (HR on admission, HR 3–6 h after admission, mucosal membranes, time of admission, PCV, and CRT) were selected for the multivariable logistic regression analysis, whose significant results are reported in Table 5. In this model, only HR 3–6 h after admission/surgery, PCV, and CRT were positively associated with the odds ratio of non-survival.

When the percentage of horses was divided into “Surgery” group (those horses that were surgically treated) and “Medical treatment“ group (those horses that received a medical treatment), they significantly differed in the outcome (Fischer Exact Test, *p* = 0.0236) (Table 6). No other differences between these groups were noted.

The surgery group was examined as a sub-dataset for the same independent variables seen for the whole selected sample. In Table 7, the signalment characteristics (body weight, age, gender, and breed) of horses belonging to the surgery group are described. No differences were observed between the discharged and non-discharged horses for these variables.

In the “Surgery” group, the variable “Distance from the surgical facility (km)” was categorized according to the distribution of data as follows: “Close”, if the distance was less than 21 km, “Medium”, if the distance ranged between 21 and 218 km, and “Far” if the distance was more than 218 km.

Table 8 describes the history data and clinical signs on admission of horses that received a surgical treatment, comparing horses discharged and non-discharged.

HR on admission, PCV, and HR 3–6 h after surgery were significantly higher in the non-discharged horses. The presence of gastric reflux was significantly more present in the non-discharged horses.

Concerning the perioperative variables (anesthetic protocol, duration of anesthesia and surgery, time to standing, administration of post-operative lidocaine, recovery score, post-operative complications, duration of hospitalization, localization, and ischemic or non-ischemic disorder), duration of anesthesia, time to standing, administration of post-operative lidocaine, duration of hospitalization, and the presence of an ischemic disorder were significantly different between discharged and non-discharged horses (Table 9).

The anesthetic protocol was categorized as follows: (1) medetomidine sedation and continuous rate infusion (CRI) of medetomidine associated with isofluorane, (2) sedation with xylazine and maintenance with isofluorane, (3) sedation with xylazine, maintenance with isofluorane and CRI of lidocaine, and (4) other which included the following associations: detomidine/butorphanol/isofluorane, and GGE/isofluorane, tiopenthal/isofluorane.

Under the variable “Site of lesion”, the category “Other” included: gastric rupture, and peritonitis.

Post-operative complications occurring were peritonitis, paralytic ileus, abdominal oedema, diarrhea, impaction, incisional hernia, laminitis, fever, jugular thrombophlebitis, left colon displacement, lidocaine neurotoxicity, abdominal adhesions, endotoxemia, and incisional infections.

As in Table 4, in Table 10 a univariate regression model is described to analyze the association between the independent variables and the outcome among the horses that received surgical treatment. HR on admission, HR at 3–6 h after surgery, PCV, duration of anesthesia, ischemic lesions, hospitalization, and time to standing were positively associated with the outcome (non-discharge). 

A multivariate regression model was built which included those horses that received a surgical treatment. As previously, those variables that showed a *p* ≤ 0.2 at the univariate model (HR on admission, HR 3–6 h after admission, PCV, PT, CRT, gastric reflux, time and month on admission, duration of anesthesia, ischemic lesion, site of the disorder, duration of hospitalization, time to standing, and mucosal membranes) were selected for the multivariable logistic regression analysis. No significant association was found.

## 4. Discussion

Medical records of the horses referred for colic syndrome were retrospectively reviewed to investigate risk and prognostic factors for the disorder. First, surgical and medical cases were analyzed together. Subsequently, those receiving a surgical treatment were reviewed separately. The same short-term outcome was considered, which was discharge from the hospital. No information was gathered about follow-up after discharge.

Colic syndrome is a well-recognized multifactorial disorder which often affects equids of every age [31]. A variety of authors have already investigated the factors associated with the manifestation of this condition and most of them are related to management condition, time spent to pasture or in the stall, or to exercise [7,8,9,11,32]. Seasonality influences type of forage and weather conditions [8,9,14,16]. For these reasons it is also acknowledged as a predisposing factor.

As these factors are all related to geographical aspects, in this retrospective study we focused on a closed cohort of horses referred to the Veterinary Teaching Hospital in Abruzzo.

The survival rate from colic syndrome is highly variable, ranging between 68% and 100%, according to the type of follow-up that was considered [33]. We observed an overall survival rate of 65% on the whole population, which is lower compared with that described by Philips and Walmsley and by Mair and Smith [34,35].

When the predisposing or risk factors were considered on the whole selected population, no difference was found between horses that were discharged and not discharged. In the univariate model, time and month of admission had a significant association with the outcome, with horses admitted between October and December more likely to be discharged than those admitted between January and March, and those admitted in the morning less likely to be discharged compared with those admitted during afternoon and evening/night.

When the same variables were considered for only the subsample of horses receiving a surgical treatment, the results were similar for the variable month but not for time on admission, which did not show an association with discharge in the univariate regression model. Regarding the seasonality, our results are in contrast with previous studies which showed a strong association of winter months with a worse outcome [17]. This difference could be ascribed to different weather conditions, frequency of weather changes, and latitude of the sites from where the horses came [13]. Moreover, for reasons related to the number of available records, we did not investigate the type of disorders occurring during each month. This can be considered a limitation of the study because it is known that some conditions are more likely to develop during specific times of the year, such as epiploic foramen entrapment, equine grass sickness, and colon torsions [36]. However, although seasonality is strongly believed to be associated with colic syndrome, many factors (stabling or exercise, feeding practice, and availability) may act as confounders, making it difficult to provide a statistical proof of this association [10,14,16,36].

Looking at the time of admission, we noticed that the worst outcome was associated with admission during the morning. This is likely due to a management reasons because horses are left alone at pasture or at the stable overnight, and clinical signs arising during late evening or night may go unnoticed and untreated until the morning, causing worsening of the conditions [2].

In addition to the predisposing factors, we also focused of the prognostic factors that were represented by all those variables potentially associated with the outcome. The characterization of the prognostic factors is essential to the veterinary surgeon to properly inform the owner of the possible outcome, to avoid unnecessary expenses to the owner and to the facility, and to accurately plan the treatment. When the whole sample was considered, significant differences were found between discharged and non-discharged horses for progression of clinical signs, mentation on admission, presence of gastric reflux, PCV, and CRT. HR on admission and HR after 3–6 h after admission or surgery, in the case of laparotomy, also significantly differed between discharged and non-discharged horses when only surgical cases were examined.

As stated by Curtis [5], the importance of pain manifestation is critical in the identification of colic syndrome, both on admission at the referral center and at first presentation of the case by the referring vet. Although scoring scales for the evaluation of pain have been developed [37], for the retrospective nature of this study, the severity of pain was only subjectively evaluated and categorized. This variable together with the progression of clinical signs from their appearance to the admission at the practice, as referred by the owner/trainer, were differently distributed in discharged and non-discharged patients, with a higher percentage of discharged horses presenting worsening of the condition despite the better mentation and cardiovascular condition. The entity of pain manifestation may reflect the degree of bowel devitalization and the severity of endotoxemia and vascular compromise [38]. Moreover, the subjective expression of pain itself may have been responsible of early recognition of the severity of the condition by the owner/trainer. Horses particularly tough to pain may have hidden the pain for longer causing a progressive deteriorating condition.

Cardiovascular variables and markers of hypovolemia on admission and after an initial stabilization were significantly higher in non-discharged horses and had a positive association with non-discharge. The importance of these variables has already been widely discussed because no matter the type of outcome and other considered condition, HR, PCV, and CRT always represent important early indicators of critical cases [2,22,39]. In addition to this, we found a significantly higher post-operative HR and an increased OR of non-discharged for higher values of HR 3–6 h after surgery. This finding was in accordance with the previous literature and can be explained by this variable’s being an indicator of endotoxemia, persistent pain, and/or hemodynamic disfunction [40,41].

Additionally, in the multivariate regression model built for the whole sample of horses, PCV and CRT were positively associated with the chances of non-discharge.

Although surgical treatment must be accompanied by a post-operative medical therapy (fluid therapy and NAIDs), it is sometimes the only choice to save the patient’s life. For this reason, we analyzed surgically treated horses separately. For them, gastric reflux, duration of anesthesia and hospitalization, time required to standing after surgery, and the presence of an ischemic lesion were also significantly associated with outcome. In detail, duration of anesthesia and of hospitalization were negatively associated with the outcome, whereas the presence of gastric reflux and the time required to standing after surgery were positively associated with it. Although longer duration of anesthesia was previously associated with worse recovery scores and worse outcome [42,43,44,45,46], we observed a longer duration of anesthesia in those horses that were discharged, likely because those patients that were euthanatized intraoperatively were included. In contrast, no significant association was found in the subset of patients that received a surgical treatment between the independent variable and outcome in the multivariate logistic regression.

Interestingly, hospitalization was longer for patients that were eventually discharged than those that did not survive, likely because the major life-threatening complications that required intensive care treatment (recurrence of colic, post-operative ileus, endotoxemia, or persistent and untreatable pain) occurred within the first days of hospitalization. This result was evident in a previous retrospective study involving a larger cohort of patients, with an average hospitalization time in an intensive care unit (ICU) of 3 days, followed by 10 days of regular stabling [47]. Moreover, Mair and Smith in 2005 observed a higher mortality rate within the first 3 days from surgery due to post-operative ileus, persistent pain and endotoxemia, which supports our hypothesis [35].

Nasogastric intubation is usually performed on admission as an adjunctive diagnostic test or to deliver enteral fluid. In this retrospective evaluation, we noticed that it was not always performed, and this could have been for unrecorded reasons (i.e., already performed by the referring vet, horse demeanor or severe pain, or absence of gastric distension at abdominal ultrasound). Although the amount of gastric reflux was not investigated, when this was found, it was significantly more represented in non-discharged horses both in the whole population and in those patients that were surgically treated. Its involvement in the univariate regression model, however, was not worthy of notice.

The time required to standing and the quality of recovery from anesthesia are known to be associated with outcome from previous studies [46,48]. New anesthetic protocols and multimodal anesthesia significantly reduced the cardiovascular and respiratory adverse effects of general anesthesia on equids, which were particularly dangerous in critically ill patients [49]. Anesthetic variables were not explored in this study, nor were the blood gases during anesthesia investigated, so we are not able to investigate their effect on the time required to standing, to the recovery score, or the outcome. We explored only the anesthetic protocol, which did not affect the outcome. Sedation with medetomidine or xylazine, with a maintenance with isoflurane and lidocaine CRI, were the most used anesthetic associations. They are both effective in reducing the minimum alveolar concentration (MAC) of inhalation agent and respiratory depression, thus preserving muscular metabolism.

The intestinal viability was subjectively evaluated during laparotomy based on serosal color and its improvement after correction of the strangulation, motility, and mesenteric arterial pulses [50]. When an ischemic lesion was suspected, enterectomy and anastomosis were considered as an option. Even though in this study a higher probability of discharge was correlated with the absence of an ischemic lesion, the macroscopic diagnosis of intestinal viability may have under- or over- estimated the disorder, as it was based on subjective evaluation. The use of objective methods to assess the intestinal viability, such as fluorescein fluorescence, thermography, surface oximetry, pulse oximetry, Doppler ultrasonography, and intraluminal pressure measurement [51,52,53,54,55] would help the surgeons to decide upon the best surgical option, although they cannot substitute the clinical judgment [56].

We also investigated the effect of transport on outcome. We supposed that a longer distance from the surgical facility would have been associated with a worse prognosis. No association was found on the whole population or on those horses that were surgically treated. This was unexpected because transport was considered as a stressful event, especially for critically ill patients [18,19,20,21]. Moreover, the time required to reach the clinic may affect the survival rate, because it increases the time from appearance of clinical signs to intervention. This could likely be due to the type of lesions that were found. Even though, for numerical reasons, only the localization of the primary disorder was investigated (small vs. large intestine), it did not differ between discharged and non-discharged horses, with a larger percentage of displacements rather than torsions.

We did not find any difference for body weight, age, gender, and breed when comparing the whole population to only horses who received a surgical treatment. These variables did not show any association with the outcome in any regression model that was built. Age was considered a “red flag” to be used to differentiate between critical versus non-critical cases [2]. Some authors identified this variable as negatively associated with survival [57,58], while others found no association [35] or identified age as a protective factor [13]. The reason for discrepancy can be related to the patients (older horses are more frequently affected by neoplasia, pedunculated lipomas, or other co-morbidities) or to the owners (keener to accept an intensive care or surgical treatment or an insurance policy on a young horse rather than an old one) [2]. The influence of body weight may be more likely related to the anesthetic risk of developing cardiocirculatory complications that were not investigated in this study. The effect of gender has been debated in literature with contrasting results likely due to the variety of conditions and different outcome that were considered. Overall, no clear association between gender and colic has been found [9,10,15,58,59,60]. An exception to this is the colon torsion which is considered associated with foaling in the mare and inguinal herniation in the stallion [7]. Concerning breed predisposition, in this retrospective study the more represented breeds (Warmbloods, Thoroughbred, and Western riding horses) were almost equally distributed in discharged and non-discharged horses. In addition, in other studies, the impact of this variable on the outcome has been discussed and results were not always consistent probably because bias by the breeds referred to the clinics or because management factors, such as exercise and diet, may play a role as confounding factors [7].

The retrospective nature of the study made it impossible to retrieve full data for all patients. The choice to include only one referral center may have biased the results and reduced the number of patients and the amount of information potentially available. Despite this limitation, our results were consistent with previous studies that identified seasonality as one of the main risk factors [16] and cardiovascular variables as the most important prognostic indicators of critical outcome, making this study an aid and a treatment guideline for the veterinary surgeon.

## 5. Conclusions

For its multifactorial etiology, a variety of risk factors for colic syndrome were previously identified. We tried to analyze risk factors in a cohort of patients, and we identified month and time of admission as associated with the outcome, showing a worse prognosis for those admitted from January to March and during the morning. Although the decision-making process for colic syndrome is multifactorial and based on owners’ personal decision, economic availability, and the horse insurance status, knowledge of prognostic indicators of outcome is mandatory for the veterinary surgeon to inform the owner and plan the most appropriate treatment. As in previous retrospective studies, in this case cardiovascular variables and markers of hypovolemia were associated with outcome. Since comparison with the previous literature is difficult because of differences in the categorization of available data, future prospective assessments in more homogenous cohorts of patients would be useful to increase consistency of the results.

## Figures and Tables

**Table 1:** Gender, age, and body weight of the selected horses. Gender is described by frequency distribution (%); age (years) and body weight (kg) are described by mean and standard deviation.

	Surgery (*n* = 82)	Medical Treatment (*n* = 75)	Total (*n* = 157)
Gender			
Gelding	25 (30%)	22 (29%)	47 (30%)
Female	29 (36%)	37 (49%)	66 (42%)
Stallion	28 (34%)	16 (22%)	44 (28%)
Age	9.73 ± 5.09	11.32 ± 5.69	10.5 ± 5.5
Body weight	467.08 ± 115.45	469.66 ± 83.9	467.29 ± 103.04

**Table 2:** Comparison of signalment findings in discharged and non-discharged horses (BW: body weight; F: female; and M: stallion).

	Discharged (*n* = 102)	Non-Discharged (*n* = 55)	*p*-Value
BW (kg)	464.6 ± 109.31	473.6 ± 96.88	0.685
Age (years)	10.3 ± 4.95	10.9 ± 6.47	0.583
Gender	F 44 (28%) M 25 (16%) Gelding 33 (21%)	F 22 (14%) M 19 (12%) Gelding 14 (9%)	0.3314
Breed	Thoroughbreds 18 (11%) Warmbloods 39 (25%) Western riding horses 25 (16%) Spanish horses 1 (1%) Friesians 5 (3%) Others 14 (9%)	Thoroughbreds 13 (10%) Warmbloods 15 (9%) Western riding horses 13 (8%) Spanish horses 2 (1%) Friesians 2 (1%) Others 10 (6%)	0.5529

**Table 3:** Comparison of history data and clinical signs on admission between discharged and non-discharged horses (Distance from the surgical facility, Progression of clinical signs, Medication received before referral, Season of admission, Time of admission, Severity of pain, Mentation, Mucosal Membranes, Gastric Reflux, and Abdominal Distension, are expressed as frequency distribution (%); PCV, TP and rectal temperature are expressed as mean ± standard deviation; HR on admission, CRT, and HR at 3–6 h after admission/surgery are expressed as median and range) (NA: not available; CRT: capillary refill time; HR: heart rate; PCV: packed cell volume; and TP: total protein).

Variable	Discharged (*n* = 102)	Non-Discharged (*n* = 55)	*p*-Value
Distance from the surgical facility (km)	Close 26 (16.5%)	Close 8 (5%)	0.6034
	Medium 47 (30%)	Medium 21 (13%)	
	Far 23 (14.5%)	Far 12 (8%)	
	NA 6 (4%)	NA 14 (9%)	
Progression of clinical signs	No progression 3 (2%)	No progression 4 (2.5%)	0.04
	Deteriorating 15 (9.5%)	Deteriorating 6 (4%)	
	Improved 8 (5%)	Improved 0 (0)	
	NA 76 (48%)	NA 45 (29%)	
Medication received before referral	NSAIDs 18 (11.5%)	NSAIDs 8 (5%)	0.8
	Mixed 50 (32%)	Mixed 25 (16%)	
	No medication 26 (16%)	No medication 13 (8.5%)	
	Spasmolytics 4 (2.5%)	Spasmolytics 2 (1.5%)	
	Crystalloids 1 (0.5%)	Crystalloids 0 (0)	
	Alpha-2 agonists 1 (0.5%)	Alpha-2 agonists 2 (1.5%)	
	NA 2 (1.5%)	NA 5 (3%)	
Season of admission	January–March 17 (11%)	January–March 16 (10%)	0.55
	April–June 33 (21%)	April–June 17 (11%)	
	July–September 26 (17%)	July–September 11 (6.5%)	
	October–December 26 (17%)	October–December 11 (6.5%)	
Time of admission	Morning 14 (9%)	Morning 10 (6.5%)	0.2447
	Afternoon 22 (14%)	Afternoon 12 (7.5%)	
	Evening–night 28 (18%)	Evening–night 9 (6%)	
	NA 38 (24%)	NA 24 (15%)	
Severity of pain	None 9 (6%)	None 0 (0)	0.07
	Mild 26 (16.5%)	Mild 8 (5%)	
	Moderate 16 (10%)	Moderate 11 (7.5%)	
	Severe 16 (10%)	Severe 10 (6.5%)	
	NA 35 (22%)	NA 26 (16.5%)	
Mentation	Bright 33 (21%)	Bright 11 (7%)	0.02
	Depressed 26 (17%)	Depressed 25 (16%)	
	Stuporous 0 (0)	Stuporous 2 (1%)	
	Agitated 9 (6%)	Agitated 0 (0)	
	NA 34 (22%)	NA 17 (11%)	
Mucosal membranes	Normal 14 (9%)	Normal 4 (2.5%)	0.07
	Pale 18 (11.5%)	Pale 5 (3%)	
	Hyperemic without toxic line 35 (22%)	Hyperemic without toxic line 24 (15.5%)	
	Hyperemic with toxic line 29 (18.5%)	Hyperemic with toxic line 16 (10%)	
	Cyanotic 3 (2%)	Cyanotic 6 (4%)	
	Brick red 3 (2%)	Brick red 0(0)	
CRT (seconds)	3 (2–3.35)	3 (2–4)	0
HR on admission (beats/min)	48 (42–65)	70 (51–91)	0.00019
Gastric reflux	Yes 14 (9%)	Yes 20 (13%)	0.0005
	No 37 (23.5%)	No 8 (5%)	
	Not performed 16 (10%)	Not performed 5 (3%)	
	NA 35 (22.5%)	NA 22 (14%)	
Abdominal distension	None 6 (4%)	None 12 (7.5%)	0.826
	Bilateral 36 (23%)	Bilateral 18 (11.5%)	
	Left 8 (5%)	Left 2 (1.5%)	
	Right 3 (2%)	Right 1 (0.5%)	
	NA 49 (31%)	NA 22 (14%)	
PCV (%)	40.86 ± 8.42	50.95 ± 12.27	0
TP (g/dL)	6.64 ± 1.23	6.77 ± 1.3	0.597
Rectal temperature (°C)	37.73 ± 0.74	37.73 ± 0.9	0.9
HR 3–6 h after admission/surgery (beats/min)	45 (40–59)	61 (52–75)	0.0002

**Table 4:** Univariate logistic regression model showing the association between the explanatory variables and the outcome (=non-discharge) in 157 horses admitted for colic syndrome at the Veterinary Teaching Hospital of Teramo (OR: odds ratio; CI: confidence interval; CRT: capillary refill time; HR: heart rate; PCV: packed cell volume; TP: total protein; and BW: body weight).

Variable	Category	Odds Ratio	95% CI	*p*-Value
Distance from the surgical facility (km)	Close (Ref) * Medium Far	1.45 1.7	0.565–3.74 0.59–4.87	0.606 ^a^ 0.439 0.327
Age (years)		1.20	0.956–1.08	0.57
HR on admission (beats/min)		1.03	1.01–1.04	0.0005
Mentation	Bright (Ref) *			0.5717 ^a^
	Depressed	0.0002	0.889–4.88	0.0913
	Stuporous	0.0007	0.000–3.70	0.9950
	Agitated	0.0000	0.533–3.86	0.9900
HR 3–6 h after admission (beats/min)		1.06	1.03–1.09	0.004
PCV (%)		1.1	1.06–1.15	0.0000
TP (g/dL)		1.090	0.7960–1.49	0.594 ^a^
Mucosal membranes	Normal (Ref) *			0.01537 ^a^
	Pale	2.25	0.2070–24.4	0.5050
	Hyperemic without toxic line	8	0.97–66.00	0.0534
	Hyperemic with toxic line	6.22	0.7320–52.90	0.0940
	Cyanotic	16	1.27–201	0.0317
	Brick red	0.00000208		0.0030
Gastric reflux	Yes (Ref) *			0.002 ^a^
	No	0.203	0.0598–0.690	0.010
	Not performed	0.000	0.0000–Inf	0.991
CRT (seconds)		2.47	1.48–4.13	0.0005
Rectal temperature (°C)		1	0.624–1.6	0.999
Progression of clinical signs	No progression (Ref) *			0.411 ^a^
	Deteriorating	0.3	0.51–1.76	0.183
	Improved	0.0000	0.00000–Inf	0.993
Gender	Female (Ref) *			0.33 ^a^
	Stallion	1.510	0.675–3.390	0.315
	Gelding	0.770	0.322–1.840	0.557
BW (kg)		1	0.9970–1	0.682
Abdominal distension	None (Ref) *			0.83 ^a^
	Bilateral	1.5	0.2750–8.19	0.640
	Left	0.750	0.0808–6.96	0.8
	Right	1	0.0625–16	1
Time of admission	Morning (Ref) *			0.00137 ^a^
	Afternoon	0.764	0.261–1.61	0.157
	Evening–night	0.450	0.149–1.36	0.991
Month of admission	January–March (Ref) *			0.05 ^a^
	April–June	0.547	0.223–1.350	0.1890
	July–September	0.450	0.168–1.2	0.110
	October–December	0.245	0.08–0.751	0.0139
Medication received before referral	NSAIDs (Ref) *	1.12	0.43.2.94	0.9290 ^a^
	Mixed	1.12	0.387–3.27	0.8290
	No medication	1.13	0.170–7.45	0.9030
	Spasmolytics	0.0000	0.0000–inf	0.9880
	Crystalloids	4.5	0.355–57.1	0.2460

* Ref: reference category for categorical predictors; the ORs for a category show the odds of non-discharge in the reference category. ^a^*:* Overall *p*-value for categorical variables with more than 2 categories.

**Table 5:** Significant results of the multivariate logistic regression model used to evaluate the association between the independent variables and the outcome (=not discharge) in 157 horses admitted for colic syndrome at the Veterinary Teaching Hospital of Teramo (PCV: packed cell volume; CRT: capillary refill time; and CI: confidence interval).

Variable	Odds Ratio	95% CI	*p*-Value
HR 3–6 h after admission/surgery (beats/min)	1.05	1.01–1.1	0.0274
PCV (%)	1.06	1.01–1.11	0.011
CRT (seconds)	1.88	1.04–3.4	0.03

**Table 6:** Percentage of horses that received a surgical (Surgery) and medical treatment (Medical Treatment) compared with the outcome (Discharged vs. Non-discharged). Statistical significance is set for *p* < 0.05.

	Surgery (*n* = 82)	Medical Treatment (*n* = 75)	Total (*n* = 157)	*p*-Value
Discharged	44 (54%)	58 (77%)	102 (65%)	0.0236
Non-discharged	38 (46%)	17 (23%)	55 (35%)	

**Table 7:** Comparison of signalment findings in discharged and non-discharged horses that received a surgical treatment (BW: body weight; F: female; M: stallion; and Mc: gelding).

Variable	Discharged (*n* = 44)	Non-Discharged (*n* = 38)	*p*-Value
BW (kg)	464.6 ± 123.9	472.7 ± 99.35	0.787
Age (years)	9.5 ± 4.41	9.4 ± 5.79	0.944
Gender	F 16 (19%)	F 13 (16%)	0.6178
	M15 (18%)	M 13 (16%)	
	Mc 13 (16%)	Mc 12 (15%)	
Breed	Thoroughbreds 8 (10%)	Thoroughbreds 6 (7.5%)	0.4205
	Warmbloods 17 (21%)	Warmbloods 10 (12%)	
	Western riding horses 13 (16%)	Western riding horses 13 (16%)	
	Spanish horses 0 (0)	Spanish horses 2 (2.5%)	
	Friesians 3 (3.5%)	Friesians 1 (1%)	
	Others 3 (3.5%)	Others 6 (7%)	

**Table 8:** Comparison of history data and clinical signs on admission between discharged and non-discharged horses that received a surgical treatment (PCV and TP are expressed as mean ± standard deviation; HR on admission, CRT, and HR at 3–6 h after admission/surgery are expressed as median and range) (NA not available; CRT: capillary refill time; HR: heart rate; PCV: packed cell volume; and TP: total protein).

Variable	Discharged (*n* = 44)	Non-Discharged (*n* = 38)	*p*-Value
Distance from the surgical facility (km)	Close 9 (11%)	Close 7 (8.5%)	0.8832
	Medium 21 (26%)	Medium 16 (19.5%)	
	Far 12 (14.5%)	Far 7 (8.5%)	
	NA 2 (2%)	NA 8 (10%)	
Progression of clinical signs	No progression 3 (3.5%)	No progression 2 (2.5 %)	0.2384
	Deteriorating 11 (13.5%)	Deteriorating 2 (2.5%)	
	Improved 5 (6%)	Improved 0 (0)	
	NA 25 (30%)	NA 34 (41%)	
Medication received before referral	NSAIDs 9 (11.4%)	NSAIDs 8 (8.9%)	0.8
	Mixed 21 (27.8%)	Mixed 16 (19%)	
	None 9 (12.7%)	None 10 (11.4%)	
	Spasmolytics 2 (3.8%)	Spasmolytics 2 (1.3%)	
	Alpha-2 agonists 3 (1.3%)	Alpha-2 agonists 2 (2.5%)	
Month of admission	January–March 5 (6%)	January–March 11 (13.5%)	0.4983
	April–June 16 (19.5%)	April–June 12 (15%)	
	July–September 10 (12%)	July–September 12 (15%)	
	October–December 13 (16%)	October–December 3 (3%)	
Time of admission	Morning 3 (3.5%)	Morning 8 (9.5%)	0.1368
	Afternoon 12 (14.5%)	Afternoon 8 (9.5%)	
	Evening–night 13 (16%)	Evening–night 8 (9.5%)	
	NA 16 (19.5%)	NA 14 (17%)	
Severity of pain	None 3 (3.5%)	None 0 (0)	0.4471
	Mild 8 (9.5%)	Mild 7 (8.5%)	
	Moderate 9 (11%)	Moderate 7 (8.5%)	
	Severe 9 (11%)	Severe 9 (11%)	
	NA 15 (18.5%)	NA 15 (18.5%)	
Mentation	Bright 15 (19.2%)	Bright 9 (11.5%)	0.4589
	Depressed 13 (16.7%)	Depressed 18 (23.1%)	
	Agitated 8 (10.3%)	Agitated 0 (0)	
	NA 8 (10.3%)	NA 7 (9%)	
Mucosal membranes	Normal 3 (3.5%)	Normal 1 (1%)	0.2222
	Pale 8 (9.5%)	Pale 2 (2.5%)	
	Hyperemic without toxic line 15 (18.5%)	Hyperemic without toxic line 13 (16%)	
	Hyperemic with toxic line 13 (16%)	Hyperemic with toxic line 12 (14.5%)	
	Cyanotic 0 (0)	Cyanotic 2 (2.5%)	
	NA 5 (6%)	NA 8 (9.5%)	
CRT (seconds)	2 s 8 (9.5%)	2 s 1 (1%)	0.07204
	3 s 24 (29%)	3 s 6 (7%)	
	4 s 12 (14.5%)	4 s 13 (16%)	
	5 s 0 (0%)	5 s 18 (22%)	
HR on admission (beats/min)	51.5 (42–67)	54 (49–87)	0.0293
Gastric reflux	Yes 5 (6%)	Yes 11 (13.5%)	0.0576
	No 12 (14.5%)	No 6 (7%)	
	Not performed 9 (11%)	Not performed 4 (5%)	
	NA 18 (22%)	NA 17 (21%)	
Abdominal distension	None 3 (3.5%)	None 2 (2.5%)	0.6457
	Bilateral 22 (27%)	Bilateral 13 (16%)	
	Left 2 (2.5%)	Left 1 (1%)	
	Right 0 (0)	Right 1 (1%)	
	NA 17 (21%)	NA 21 (25.5%)	
PCV (%)	39.7 ± 6.23	49.8 ± 12.16	0.004
TP (g/dL)	6.5 ± 1.00	6.95 ± 1.1	0.106
Rectal temperature (°C)	37.6 ± 0.77	37.5 ± 0.77	0.709
HR 3–6 h after admission/surgery (beats/min)	50 (42–60)	60 (52–75)	0.0296

**Table 9:** Perioperative variables of the Surgery group according to the outcome. Duration of anesthesia, time to standing, administration of post-operative lidocaine, duration of hospitalization, and the presence of an ischemic disorder was significantly different between discharged and non-discharged horses. Statistical significance was set at *p* < 0.05.

Variable	Discharged (*n* = 44)	Non-Discharged (*n* = 38)	*p*-Value
Anesthetic protocol	(1) 17 (21%) (2) 13 (16%) (3) 5 (6%) (4) 4 (5%) NA 5 (6%)	(1) 15 (18%) (2) 9 (11%) (3) 0 (0) (4) 3 (3.5%) NA 11 (13.5%)	0.2676
Duration of surgery (min)	91.2 ± 42.12	89.4 ± 38.47	0.871
Duration of anesthesia (min)	127.7 ± 36.19	99 ± 41.64	0.0122
Time to standing (min)	45 (35–77.5)	90 (62–110)	0.027
Post-operative Lidocaine infusion	Yes 5 (6%) No 37 (45%) NA 2 (2.5%)	Yes 7 (8.5%) No 15 (18%) NA 16 (20%)	0.05255
Recovery score	(0) 1 (1%) (1) 5 (6%) (2) 1 (1%) (3) 2 (2.5%) (4) 4 (5%) (5) 13 (16%) (6) NA 18 (22%)	(0) 0 (0) (1) 2 (2.5%) (2) 2 (2.5%) (3) 2 (2.5%) (4) 0 (0) (5) 4 (5%) (6) NA 28 (34%)	0.3776
Post-operative complications	Yes 16 (19.5%) No 28 (34%)	Yes 18 (22%) No 20 (24.5%)	0.5698
Hospitalization (days)	9.5 (7–13.25)	1 (0–5.25)	0.0000
Localization of the disorder	Small intestine 12 (14.5%) Large intestine 31 (38%) Others 1 (1%)	Small intestine 8 (10%) Large intestine 22 (27%) Others 5 (6%) NA 3 (3.5%)	0.09443
Ischemic/non-ischemic lesion	Yes 3 (3.5%) No 39 (47%) NA 3 (3.5%)	Yes 10 (12%) No 15 (18%) NA 13 (16%)	0.001

**Table 10:** Univariate logistic regression model showing the association between the explanatory variables and the outcome (=non-discharge) in 82 horses that received a surgical treatment for colic syndrome at the Veterinary Teaching Hospital of Teramo (OR: odds ratio; CI: confidence interval; CRT: capillary refill time; HR: heart rate; PCV: packed cell volume; TP: total protein; and BW: body weight).

Variable	Category	Odds Ratio (OR)	95% CI	*p*-Value
Distance from the surgical facility (km)	Close (Ref) *			0.88 ^a^
	Medium	0.750	0.193–2.92	0.678
	Far	0.980	0.3–3.20	0.973
Age (year)		0.997	0.911–1.09	0.943
HR on admission (beats/min)		1.02	1.00–1.04	0.0301
HR 3–6 h after admission/surgery (beats/min)		1.04	1–1.080	0.0461
Mentation	Bright (Ref) *			0.5155 ^a^
	Depressed	2.31	0.775–6.88	0.133
	Stuporous	0.0000	0.000–inf	0.9900
	Agitated	1.46	0.394–5.4	0.572
PCV (%)		1.13	1.05–1.21	0.0006
PT (gr/dL)		1.56	0.9–2.72	0.1120
Mucosal membranes	Normal (Ref) *			0.5852 ^a^
	Pale	0.750	0.0347–3.2	0.837
	Hyperemic without toxic line	2.6	0.0483–11.6	0.432
	Hyperemic with toxic line	2.770	0.2520–30.4	0.405
CRT (seconds)	2 s (Ref) *			0.0684 ^a^
	3 s	0.324	0.0868–1.2	0.0924
	4 s	0.5	0.125–2	0.3270
	5 s	0.115	0.0221–0.602	0.0104
Rectal temperature (°C)		0.878	0.448–1.6	0.704
Gastric reflux	Yes (Ref) *			0.0686 ^a^
	No	0.227	0.0538–0.961	0.0440
	Not performed	0.202	0.0415–0.983	0.0476
Progression of clinical signs	No progression (Ref) *			0.5528 ^a^
	Deteriorating	0.273	0.0263–2.83	0.276
	Improving	0.0000	0.00000–Inf	0.995
Gender	Female (Ref) *			0.6207 ^a^
	Stallion	1.070	0.376–3.03	0.903
	Gelding	0.615	0.192–1.97	0.414
BW (kg)		1	0.9976–1.01	0.783
Abdominal distension	None (Ref) *			0.998 ^a^
	Bilateral	0.886	0.13–6.02	0.640
	Left	0.750	0.0376–15	0.8
	Right	1	0.0625–16	1
Time at admission	Morning (Ref) *			0.1615 ^a^
	Afternoon	0.273	0.0263–2.83	0.0895
	Evening–night	0.0003	0.0000–inf	0.0711
Month on admission	January–March (Ref) *			0.068 ^a^
	April–June	0.324	0.0869–1.2	0.0924
	July–September	0.5	0.1250–2	0.3270
	October–December	0.115	0.0221–0.2 ^a^	0.0104
Medication received before referral	NSAIDs (Ref) *			0.8483 ^a^
	Mixed	0.877	0.2680–2.87	0.828
	None	1.16	0.3040–4.40	0.831
	Spasmolytics	0.429	0.0363–5.06	0.501
	Alpha-2 agonists	2.570	0.1920–34.5	0.476
Anesthetic protocol	(1) (Ref) *			0.978511 ^a^
	(2)	0.785	0.262–2.35	0.665
	(3)	0.0000	0.000–Inf	0.992
	(4)	0.85	0.163–4.43	0.847
Duration of anesthesia (min)		0.98	0.964–0.997	0.0179
Duration of surgery (min)		0.999	0.985–1.01	0.868
Ischemic lesion	Yes (Ref) *			0.0674 ^a^
	No	0.115	0.0279–0.478	0.0029
Post-operative lidocaine	Yes (Ref) *			0.556 ^a^
	No	0.29	0.0793–1.06	0.0607
Site of the disorder	Small Intestine (Ref) *			0.160079 ^a^
	Large intestine	1.42	0.462–4.36	0.5410
	Other	10	0.944–106	0.0559
Post-operative complications	Yes (Ref) *			0.724
	No	0.769	0.311–1.9	0.570
Recovery score		0.868	0.568–1.33	0.511
Hospitalization (days)		0.627	0.506–0.778	0.000211
Time to standing (min)		1.03	1–1.05	0.0276

* Ref: reference category for categorical predictors, the ORs for a category show the odds of non-discharge in the reference category. ^a^: Overall *p*-value for categorical variables with more than 2 categories.

## Data Availability

The data presented in this study are available on request from the corresponding author. The data are not publicly available due to ethical concerns.
